# The antibody titers to Helicobacter pylori in 7 - 12 year old iron deficiency anemic children, in Ilam

**Published:** 2010

**Authors:** Morteza Hoseinzadeh, Afra Khosravi, Koroush Sayemiri, Mohammad Hossein Rasoli, Alireza Mohaveri

**Affiliations:** a Immunology, Ilam University of Medical Sciences, Ilam, Iran; b Immunology, Ilam University of Medical Sciences, Ilam, Iran; c Medical Statistics, Ilam University of Medical Sciences, Ilam, Iran; d Clinical Department, Ilam University of Medical Sciences, Ilam, Iran

**Keywords:** Anemia, Iron Deficiency, Helicobacter Pylori, Ferritins

## Abstract

**BACKGROUND:**

It has recently been revealed that H. pylori infection is one the most important causes of anemia inhibiting iron uptake. The current study was designed to evaluate the correlation between the iron deficiency anemia and IgG to H. pylori in anemic children.

**METHODS:**

In this analytical study, 100 anemic children were analyzed using total Iron, Ferritin, TIBC and H. pylori IgG assay. Data were collected using a questionnaire including parameters of age, blood group, infancy nutrition, iron consumption, fatigue, weakness, height, weight, gastrointestinal infectious, parasitic and blood diseases, parent literacy, income, inhabitation, etc. Data were analyzed using Multivariate Regression Analysis Models, Pearson Correlation- test and Kolmogrov Smirnov.

**RESULTS:**

The most prevalent blood group detected in the study sample was group O (62%); 79% were breastfed, 9% were bottle- fed, 12% were both breastfed and bottle- fed. The history of gastrointestinal disorders was mentioned amongst 91% of the patients’ family members. A significant relationship was observed between the iron level with serum, ferritin, level of TIBC and elevated level of IgG titer to H. pylori (p < 0.001). There was a significant association between the shared dishes, GI disorders, fatigue and weakness and level of TIBC, ferritin, Iron and IgG (p < 0.001).

**CONCLUSIONS:**

The significant relationship between the iron level, IgG titer and H. pylori infection rate can be referred to as important factors influencing the anemia rate. Therefore, H. pylori IgG test can be checked for anemia together with the other routine tests.

The iron deficiency anemia is one of the most common forms of anemia worldwide, mostly in children and pregnant women. This term is applied to inadequate access to iron during heamatopoesis in bone marrow. Usually about 0.8- 1.5 mg iron should be obtained from daily meals during childhood in order to make a positive balance in the body. As 10% of necessary iron can be ab sorbed from a mixed daily meal, a desired diet should include 8- 15 mg iron daily.^1^,^2^ In the early years of age, it is not possible to acquire the required amount of iron from foods.

Iron is adsorbed in proximal part of small intestine as transferrin- attached iron. The RBC progenitors can acquire the iron through transferring receptors as the mediators of this reaction. The iron, then, is released and placed inside the haem. Outside of the hemoglobin progenitor cells, iron is stored inside the ferritin. Iron from either food or stored resources are circulating in plasma.^3^ The iron deficiency is a situation in which the disposed amount of iron is more than the adsorbed amount. The first sign of this situation is the negative balance of iron. In such a situation, the stored amount of iron is decreased, depleting the plasma ferritin resulting in an increase in TIBC, which is called iron deficiency. When the stock of iron is depleting, the concentration of plasma iron is decreasing and transferrin binding level is dropped to less than 0.15% resulting in an increase in RBC protoporphyrin, which is called iron-deficiency hematopoesis. The third stage of anemia is the iron deficiency phase, which is firstly normocytic normochrome and then gradually progresses into microcytic hypochrome.^3^,^4^

The level of serum iron (SI), which is normally 5- 160 mg/dl will be decreased in anemia, while the TIBC level, which is 250- 400 mg/dl will be considerably increased. The amount or proportion of iron to TIBC is called the TIBC saturation rate, which is normally 20% to 55% and less than 15% is recognized as a sign of iron deficiency syndrome.^3^,^4^ Although there is excess amount of iron, the access to iron is low as it is relatively insoluble. Usually, the most amount of iron is as insoluble salt and the gastric acidity can help it to be adsorbed. Every factor increasing the gastric pH can decrease the iron intake. Some parameters such as quick growth during infancy and early childhood, pregnancy, taking erythropoietin, chronic bleeding, menstruation, acute bleeding, inadequate food elements, malabsorption due to auto immune disease such as crown disease, malabsorption due to surgery, acute and chronic inflammation and some environmental parameters can also decrease the iron intake.^4^

Recently it has been demonstrated that H. pylori can reduce the intake of iron in digestive system as the H. pylori-induced gastritis can decrease the gastric acidity and hence the iron deficiency.^3^–^6^ H. pylori is a flagellate bacillus from spirillacea family. There are some species of helicobacter genus recognized in the gastrit of mammalians amongst which only H. pylori can infect human being.^6^–^10^

H. pylori is a gram negative, bent, spiral, non- sporic, having 4- 6 shielded flagella, motile, with a smooth external cell wall together with glycocalix.^11^ It has a somatic antigen, heat resistant lipopolysacharide, flagellum- related heat sensitive antigen and urease antigen at the external surface and periplasmic space^12^–^14^ Initially, bacterium was sensitive to metronidazole while at present 40% of cases are resistant.^15^,^16^ This bacterium secrets different enzymes such as catalase, protease, phospholypase C, A2 gastric acid inhibitor protein, leukotactic factors, haemolysine, HSP (Heat Shock Proteins), cag A, urease, alcohol hydrogenase, PAF (Platelet Activity Factor), and gastric ulcer- induced factor^17^–^21^ The role of this bacterium in acute and chronic gastritis peptic ulcer and gastric adenocarcinoma has been proved.^22^ H. pylori can inhibit gastric acid secretion and increases the pH, resulting in the decrease of iron intake. Many researches analyzed the relationship of H. pylori infection and iron deficiency anemia. The first case report was by Dofour et al in 1993 on a child with H. pylori infection and anemia who was cured after treatment of H. pylori and without using any complementary foods and iron.^6^ Referring to such studies, which show the correlation between H. pylori and anemia and since there is few studies on this problem in Iran, this study was designed to evaluate such correlation in order to help physicians considering this infection once encountering the anemia.

## Methods

A hundred children aged 7- 12 years (68% girls and 32% boys) with definite diagnosis of iron deficiency anemia (using Human Ferritin ELISA Kit, Cat # KA0211 V.02) were randomly selected from anemic children in Ilam in 2008. Sera iron, ferritin, TIBC and IgG to H. pylori was assessed using ELISA according to the modification of method described by Voller et al.^23^ A questionnaire including parameters such as age, sex, weight, height, blood group, monthly income, parents literacy, number of children, type of nutrition in infancy, shared dishes, gastrointestinal and blood infection, decreased appetite, nausea and vomiting, stool color and consistency, reflux, fatigue and weakness, cracked lips, nail malformation, nerves disorders, irritability, bleeding, history of infections and parasitic disease was completed for each individual. Sample size was computed using α = 5% and absolute error equal to 0.22 for correlation with Acceptable Absolute Precision Formula (AAPF). A p- value of 0.05 was considered statistically significant. Data were analyzed using Pearson Correlation Test, Multivariate Regression Analysis Model, ANOVA and T- test.

## Results

Majority of anemic patients (62%) had blood group O and 89% were Rh positive. Most patients were from parents with low monthly income (69%) and with primary or secondary school literacy (78%). Up to 79% were breast feeder and 37 of those who had parents with low monthly income were exclusively breast feeders. Height analysis showed that there was no one under the 5 percentile. In other words, 77% were between percentiles of 50- 95. 55% of parents without high education literacy had children between 50- 45 percentile. The height for 56% of those who were exclusively breast feeder at the first 6 months of age was within the range of 50- 95 percentiles. The weight assessment results indicated that up to 42% of children were in 5- 50 percentiles while 58% were placed in 50- 95 percentile. Totally 52% of all infants consumed iron drops at breastfeeding period. On the other hand, 33% of those who were just breastfed, consumed iron drops too. Shared dishes was used by 37% of patients mostly those with non- literate parents. Although the risk of H. pylori infection was increased for children who washed their hand before eating and for those used shared dishes as two risk factors of H. pylori infection, surprisingly these two risk factors were more frequent amongst children whose parents were non- literate. Gastrointestinal disorders were observed amongst 17% of all studied children. Evaluation of GI symptoms showed that decreased appetite (54%), nausea (38%), reflux (36%) and fatigue (16%) were the most prevalent symptoms. It was also noteworthy that 78% had infectious or parasitic disease. Data on the amounts of ferritin, iron and TIBC against IgG level in sera using ANOVA are summarized in table 1. There was a significant difference between the decreased levels of sera iron (SI) and ferritin with increased level of TIBC, and between the decreased levels of SI, ferritin, increased levels of TIBC with the increased level of IgG to H. pylori (p = 0.001).

**Table 1:** Correlation of IgG titer to H. pylori with iron, TIBC and ferritin levels

Lab indicators		Ferritin levels	Iron levels	TIBC levels	IgG levels
Ferritin levels	Pearson	1	0.942 *	-0.970 *	-0.951 *
	correlation				
	Sig. (2-tailed)	-	0.000	0.000	0.000
	n	100	100	100	100
Sera iron levels	Pearson	0.942	1	-0.930	-0.889
	correlation				
	Sig. (2-tailed)	0.000	-	0.000	0.000
	N	100	100	100	100
TIBC levels	Pearson	-0.970	-0.930	1	0.940
	correlation				
	Sig. (2-tailed)	0.000	0.000	-	0.000
	N	100	100	100	100
IgG levels	Pearson	-0.951	-0.889	0.940	1
	correlation				
	Sig. (2-tailed)	0.000	0.000	0.000	-
	n	100	100	100	100

About 80% of children with iron deficiency anemia had an IgG titer to H.pylori, indicating a common infection; but only 10% had an IgG titer indicating invasive form of H. pylori infection.

Results of laboratory indicators with shared dishes, GI disorders, fatigue and weakness, are summarized in table 2. T- test showed a significant correlation between the sera ferritin, iron, TIBC and IgG to H. pylori with some variables such as shared dishes, GI disease, fatigue and weakness (p = 0.000). Also GI with decreased level of sera ferritin and increased level of TIBC showed a significant correlation while fatigue and weakness was significantly correlated with all lab indicators.

**Table 2:** Correlation between the shared dishes, GI disorders, fatigue and weakness with IgG, iron levels, TIBC and ferritin

Lab indicators	Shared dishes	n	Mean	Std. deviation	P value
Ferritin level	Yes	37	10.7838	1.18755	0.000
	No	63	11.9444	1.38887	-
Iron level	Yes	37	16.8595	2.76330	0.000
	No	63	19.3143	3.36082	-
TIBC	Yes	37	411.2	10.45317	0.000
	No	63	400.1	13.40423	-
IgG	Yes	37	38.3514	9.45814	0.000
	No	63	30.6349	8.90143	-


**Lab indicators**	**GI disorder**	**n**	**Mean**	**Std. deviation**	**P value**

Ferritin level	Yes	38	10.7895	1.30359	-
	No	62	11.9597	1.32213	0.000
Iron level	Yes	38	16.7842	2.84961	0.000
	No	62	19.4000	3.27434	-
TIBC	Yes	38	410.2	11.64506	0.000
	No	62	400.3	13.12834	-
IgG	Yes	38	38.0263	9.47682	0.000
	No	62	30.7097	8.99615	-

**Lab indicators**	**F and W**	**n**	**Mean**	**Std. deviation**	**P value**
	
Ferritin level	Yes	16	10.0125	1.16326	0.000
	No	84	11.8012	1.29163	-
Iron level	Yes	16	15.2438	2.05847	0.000
	No	84	19.0083	3.22196	-
TIBC	Yes	16	418.3	8.80696	0.000
	No	84	401.5	12.51071	-
IgG	Yes	16	42.6250	9.64624	0.000
	No	84	31.7500	8.87378	-

F = Fatigue, W = Weakness

**Lab indicators**	**Loss of appetite**	**n**	**Mean**	**Std. deviation**	**P value**
	
Ferritin level	Yes	54	11.0815	1.31638	-
	No	46	12.0239	1.39733	0.001
Iron level	Yes	54	17.4963	2.99257	0.003
	No	46	19.4739	3.47649	-
TIBC	Yes	54	4.0794E2	12.11956	0.002
	No	46	3.9976E2	13.75207	-
IgG	Yes	54	36.2037	9.73300	0.002

## Discussion

According to the results of this study, the O blood group (62%) and the Rh^+^ (89%) were the most prevalent group with H. pylori infection. Although some studies evaluated the relationship of H. pylori infection and blood group, majority found no significant correction.^24^–^26^ Some researchers such as Kanbay et al 2005 reported that the O and A blood group individuals were more susceptible to active H. pylori infection compared to other groups.^27^ Even though in this study it was concluded that those patient who had blood groups O and Rh^+^ were more anemic than others, there was no significant correlation between the blood group and increased level of antibody titer to H. pylori. Some studies reported the higher prevalence of iron deficiency in females^28^ while some others reported that in male infants.^29^ In the present study, it was concluded that the iron deficiency anemia was more prevalent in female children of age 7- 12 years compared to males. The result of this study showed a reverse correlation between parents’ literacy and monthly income with iron deficiency anemia, so that anemia was higher in 34% children of illiterate parents and 44% children of parents with high school and lower education compared to others. These results were indicated by other studies as well.^30^,^31^ Breastfeeding was revealed to have a reverse correlation with the anemia in the present study, as it was reported by other studies too. However, controversial results are reported; some expressed the reverse correlation,^32^ while others reported direct correlation between being only breastfed at the first 6 month of life and prevalence of anemia.^33^,^34^

As it was stated earlier in this paper, lower percentiles of height and weight was clearly seen among anemic children. Choe et al showed a decreased mean height in children with anemia compared to those without anemia.^35^ Some other studies showed that children who had iron deficiency were more susceptible to obesity.^36^ However, another study reported that iron deficiency in mothers can lead to low birth weight, immaturity, anemia and even death of the infants.^37^ The present study showed that iron drop consumption was diversely correlated with parents’ literary; however, there was no significant correlation between these two variables, probably due to some socio-cultural conditions, since majority of children with iron deficiency anemia had not consumed any iron drops.^38^

In addition, looking at variables of shared dishes and washing hands before food serving, it can be seen that parents’ literacy directly influenced the prevalence of H. pylori infection. GI symptoms were more common among anemic children who were not breastfed, so that 63% of children who were not just breastfed had no sign of GI. On the other hand, GI symp toms were diversely correlated with parents’ literacy.

It is also worthy to mention that decreased weight and appetite, nausea and vomiting, reflux and stomachache were the most common signs observed indicating the H. pylori infection. The increased levels of IgG to H. pylori was adversely proportional to sera ferritin and iron levels and directly correlated with TIBC levels as was shown by Berg et al and Kearney, too.^39^,^40^ Using shared dishes and decreased levels of sera iron, ferritin and increased levels of TIBC together with increased titer of IgG to H. pylori can be interpreted as the sign of H. pylori infection and impact on iron intake, and hence the anemia.

Generally speaking, lower levels of iron, ferritin, and higher levels of TIBC and IgG to H. pylori were clearly observed in anemic children. Similarly, Baggett et al in a research conducted in Alaska on children with H. pylori infection, suggested that H. pylori- induced chronic gastritis can independently have significant correlation with iron deficiency anemia and decreased levels of sera ferritin, which in turn indicates that active H. pylori infection can be an important risk factor for inhibition of iron intake resulting in anemia.^41^

## Conclusions

Results of this study indicated that active infection of H. pylori clearly is coincident with the decreased absorption levels of iron in children, causing iron deficiency anemia. Therefore, curing the H. pylori infection particularly in breastfed children can remove the inhibitory role of this bacterium, clearing the background of malnutrition and hence increasing the iron absorption. It is strongly recommended that baby clinics and physicians pay special attention to this infection prior to treatment protocol for anemic children.
